# Supplementary material for: 3D printable strong and tough composite organo-hydrogels inspired by natural hierarchical composite design principles
Source: Nat Commun. 2024 Apr 15;15:3237. doi: 10.1038/s41467-024-47597-7 (PMC11018840; doi:10.1038/s41467-024-47597-7)
Supplement: Supplementary file 1 — Supplementary Information [file 41467_2024_47597_MOESM1_ESM.docx]

Supplementary Information

**3D printable strong and tough composite organo-hydrogels inspired by natural hierarchical composite design principles**

*Quyang Liu, Xinyu Dong, Haobo Qi, Haoqi Zhang, Tian Li, Yijing Zhao, Guanjin Li, Wei Zhai**

Department of Mechanical Engineering, National University of Singapore, 9 Engineering Drive 1, 117575, Singapore.

* Corresponding author. E-mail: mpezwei@nus.edu.sg

**
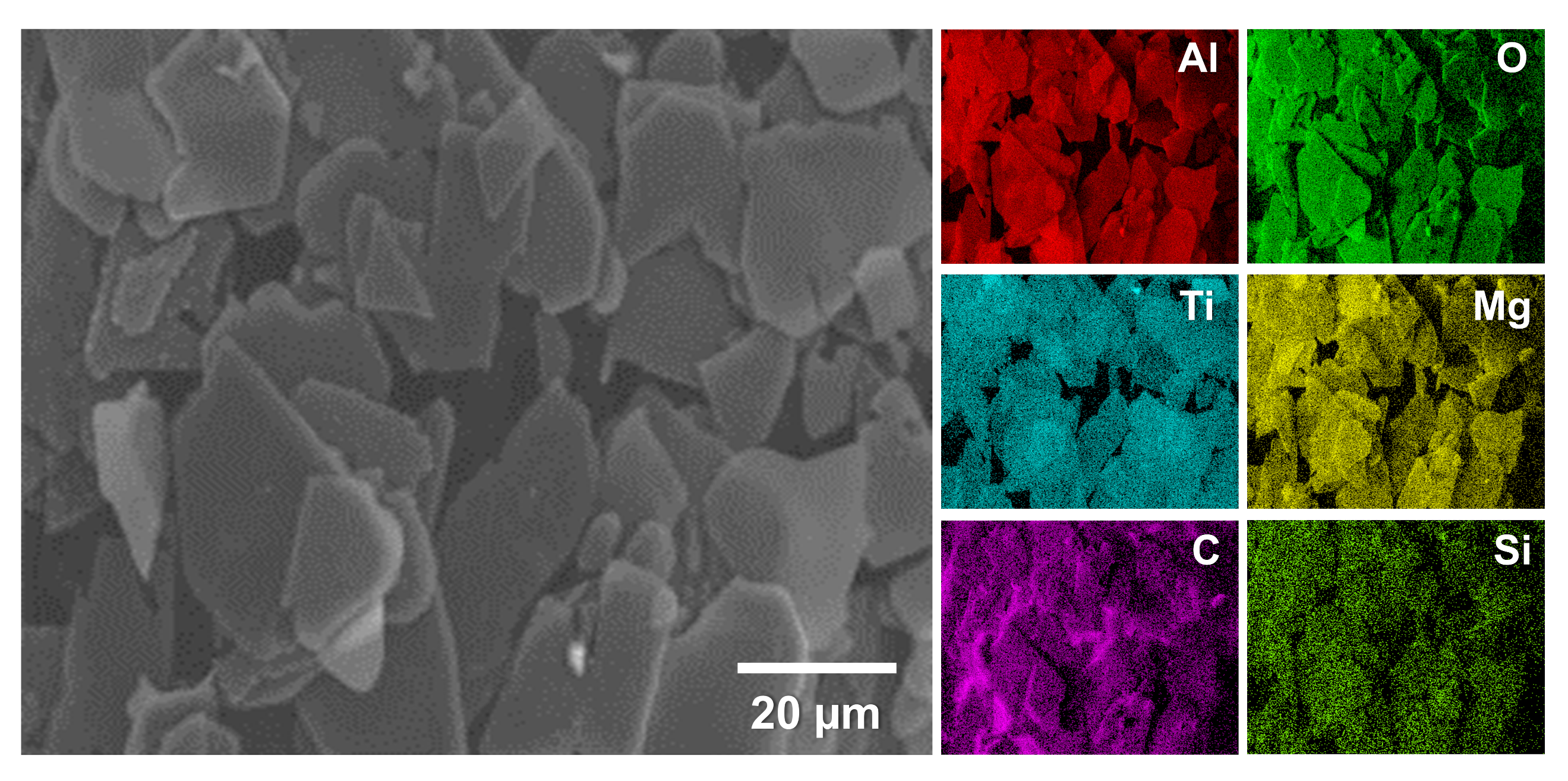

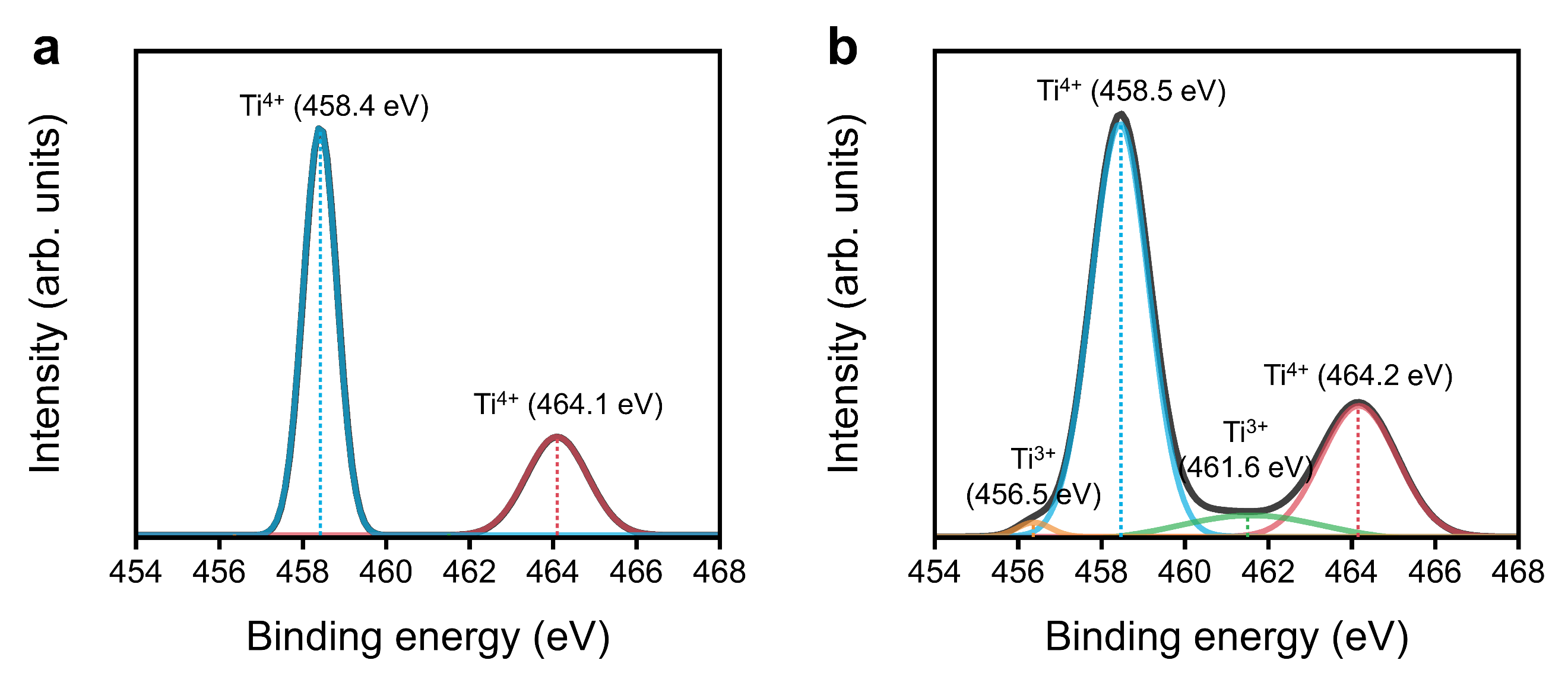
Supplementary Fig. 1.** Ti 2*p* X-ray photoelectron spectroscopy (XPS) of the ceramic microplatelets **a**) before and **b**) after Mg-thermic reduction, indicating the formation of Ti^3+^ (with peaks at 456.5 eV and 461.6 eV) in addition to Ti^4+^ (at 458.5 eV and 464.2 eV) in the conductive ceramic microplatelets.

**Supplementary Fig. 2.** Energy dispersive X-ray spectroscopy (EDX) mapping of pre-treated ceramic microplatelets indicating the presence of Mg (a result of Mg^2+^ doping of the titania during Mg-thermic reduction) and Si (due to surface modification with APTES) elements.

**
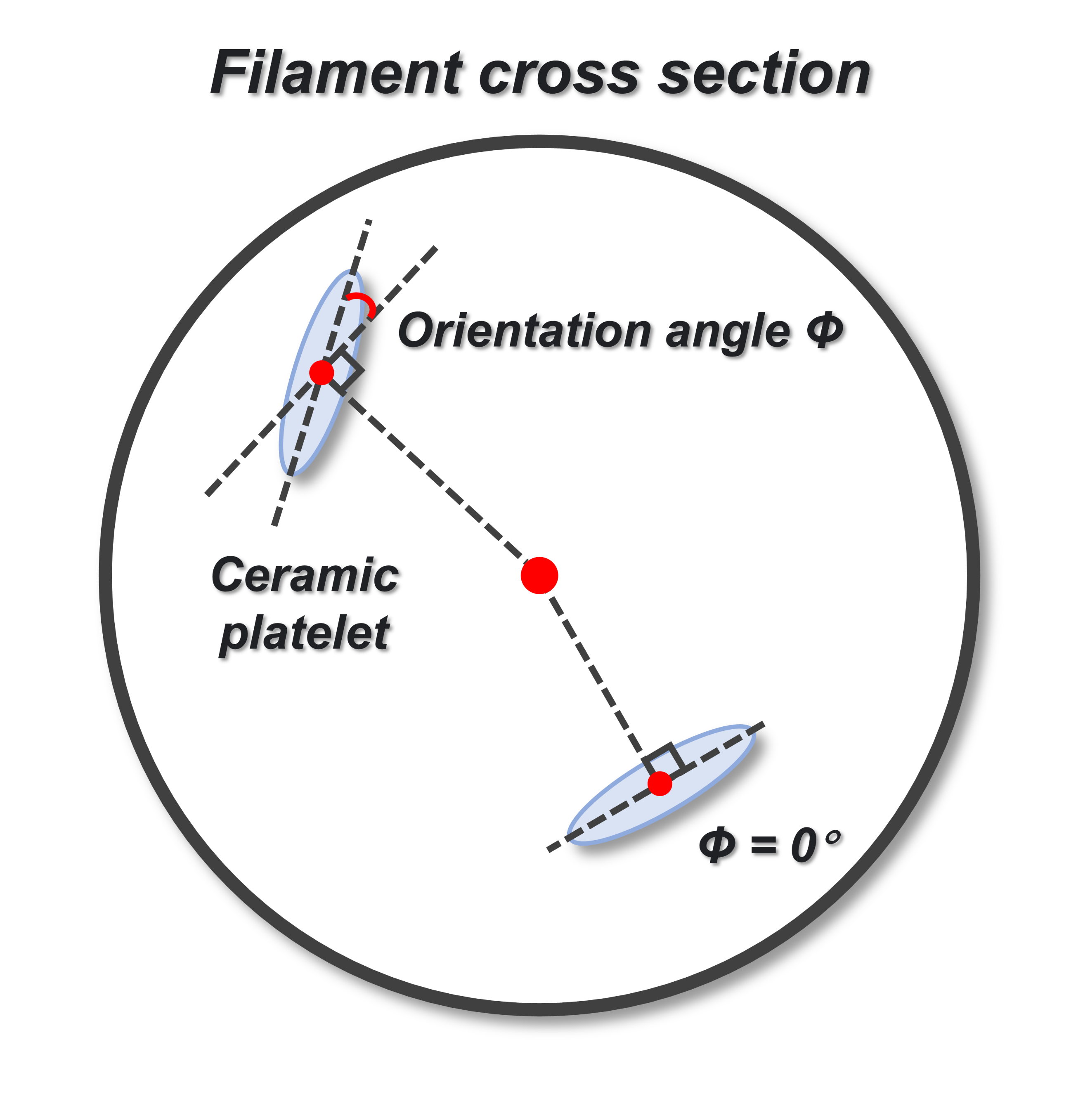
Supplementary Fig. 3.** Schematic illustration of the definition and calculation of the orientation angle Φ of ceramic platelets with respect to the center of the filament cross section.

**Supplementary Fig. 4.** Tensile stress-strain curve of pure PVA hydrogel upon freeze-thawing twice.

**
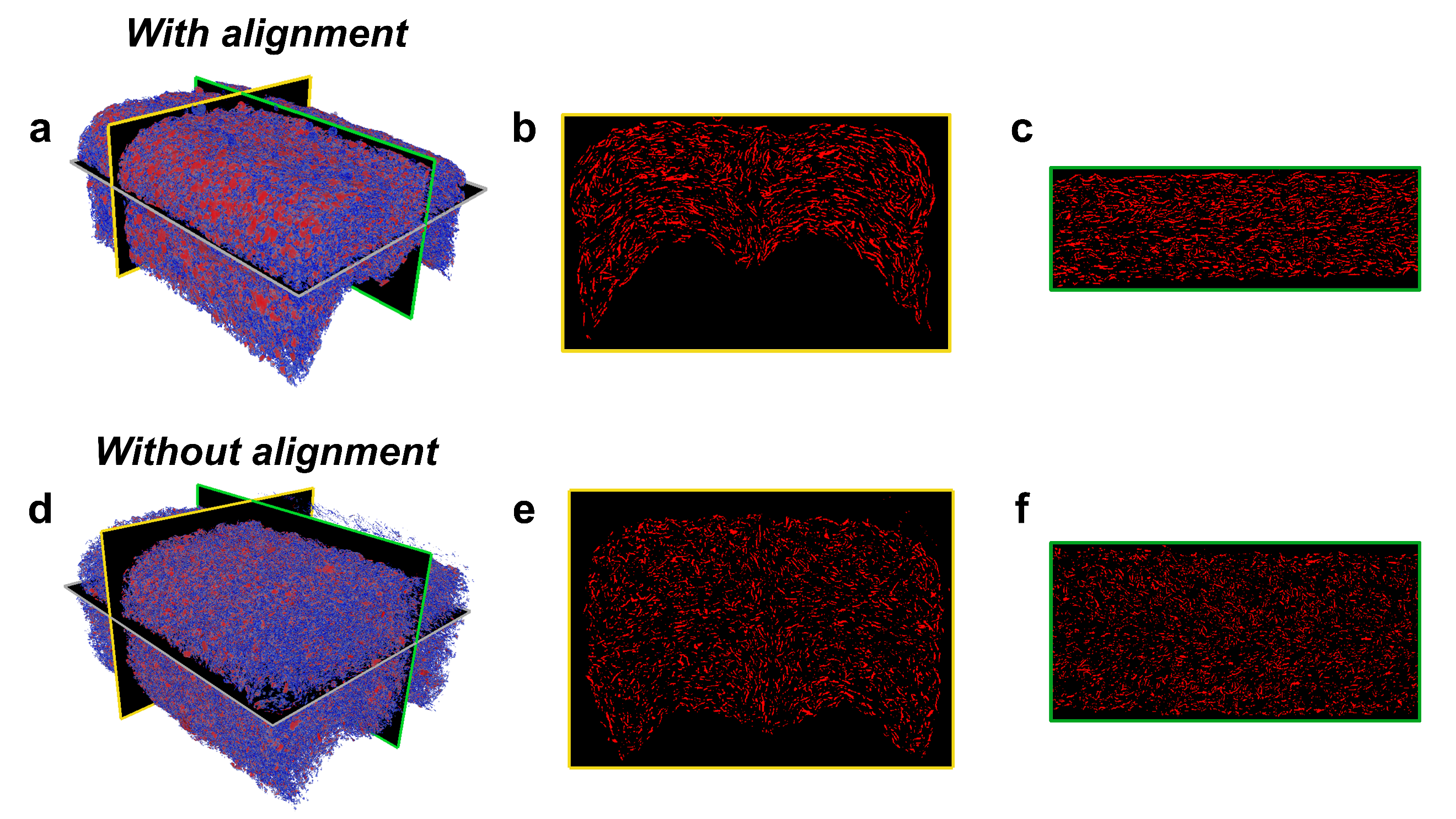
**

**Supplementary Fig. 5.** CT scans of composite organo-hydrogel samples formed by 2x2 parallel filaments with **a-c**) aligned and **d-f**) randomly distributed ceramic platelets, including captures of transverse (middle) and longitudinal (right) views.

In the top panel (Supplementary Fig. S5a-c), more pronounced platelet alignment can be observed in both transverse and longitudinal views. The scans also indicate good adhesion in between individual filaments. It should be noted that shape distortions are evident in these samples due to drying. In particular, samples with aligned platelets demonstrate more significant distortions, which is also associated with their internal microstructure of aligned platelets.


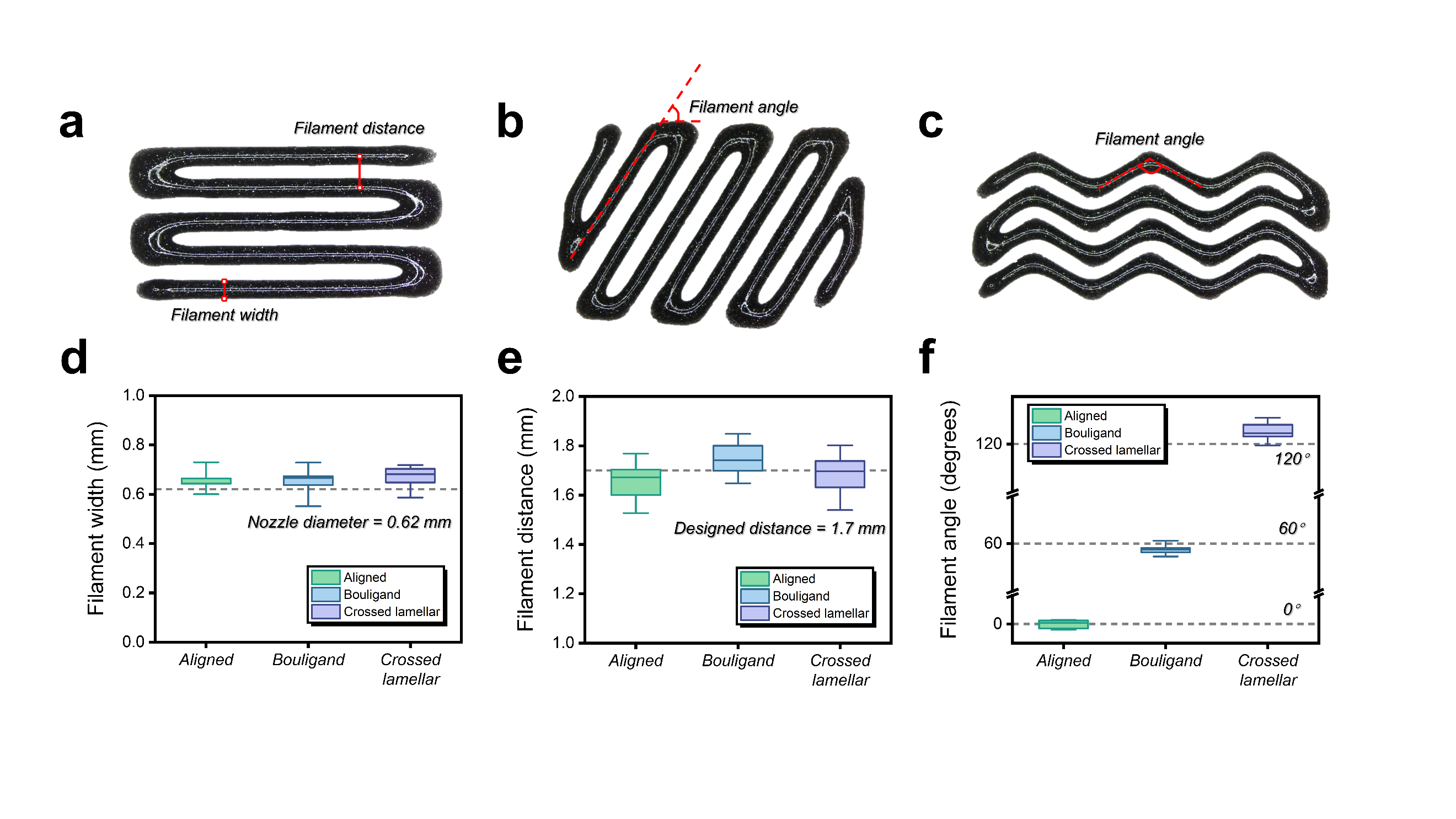


**Supplementary Fig. 6.** Precision of DIW 3D printing with composite ink across different bioinspired infill patterns. **a-c**) Optical images of printed patterns in aligned, Bouligand, and crossed lamellar samples, respectively. **d-f**) Comparison of the printed filament width, distance, and angle against the designed parameters, respectively. The box plots indicate median by the middle line, 25th and 75th percentile by the box bounds, and 5th and 95th percentile by the whiskers.

**
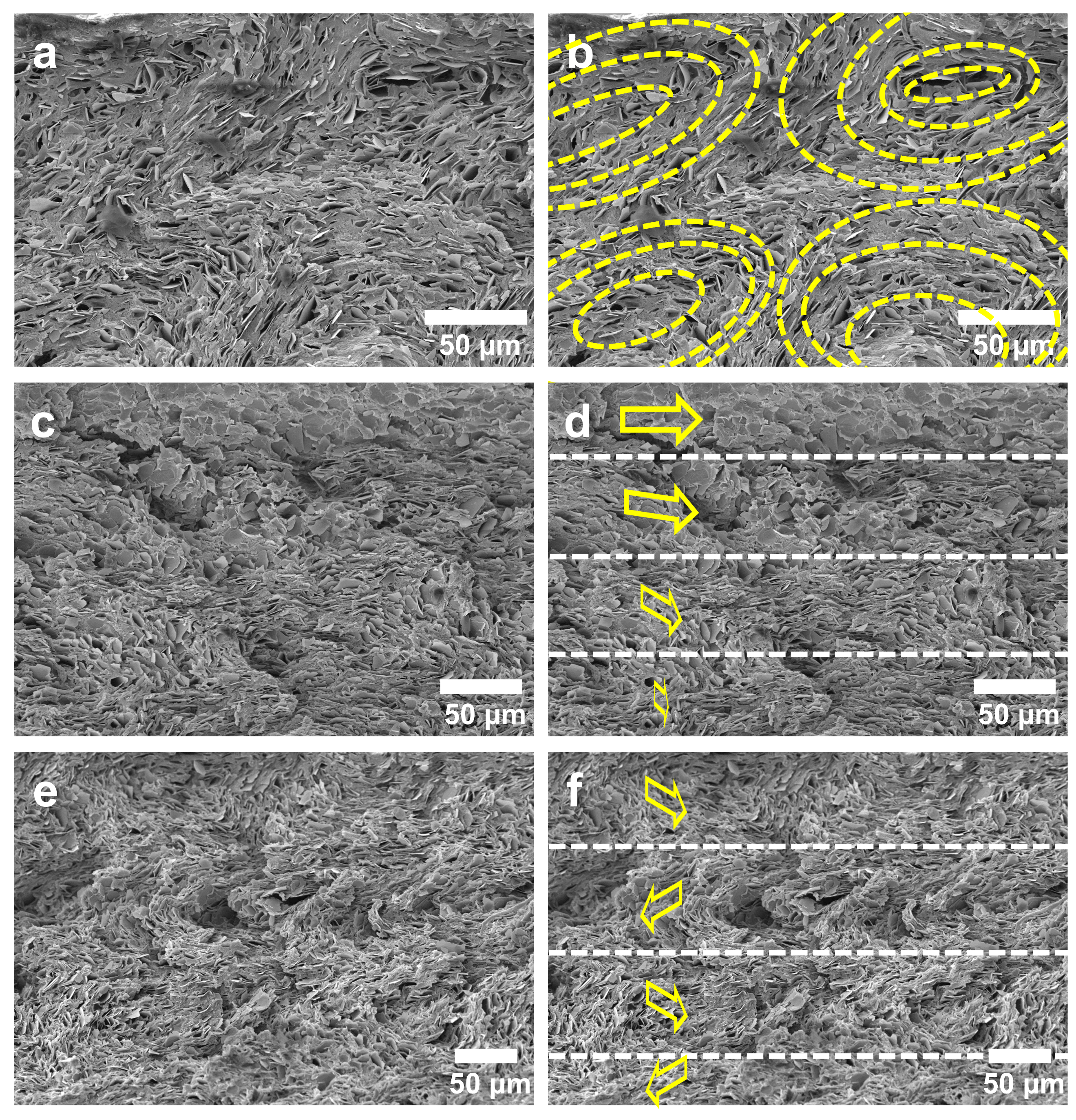
Supplementary Fig. 7.** SEM images of the cross section of 3D printed composite organo-hydrogels with **a-b**) aligned, **b-c**) Bouligand, and **c-d**) crossed lamellar macro-architectures. Platelet orientations across the layers are marked in the right panel.

In the unidirectionally aligned sample, platelets are aligned into to the viewing plane. Both Bouligand and crossed lamellar samples showed varying platelet orientations: the Bouligand sample exhibits varying degrees of platelet orientation across the layers, while the crossed lamellar sample shows alternating platelet orientations in two adjacent layers.

**
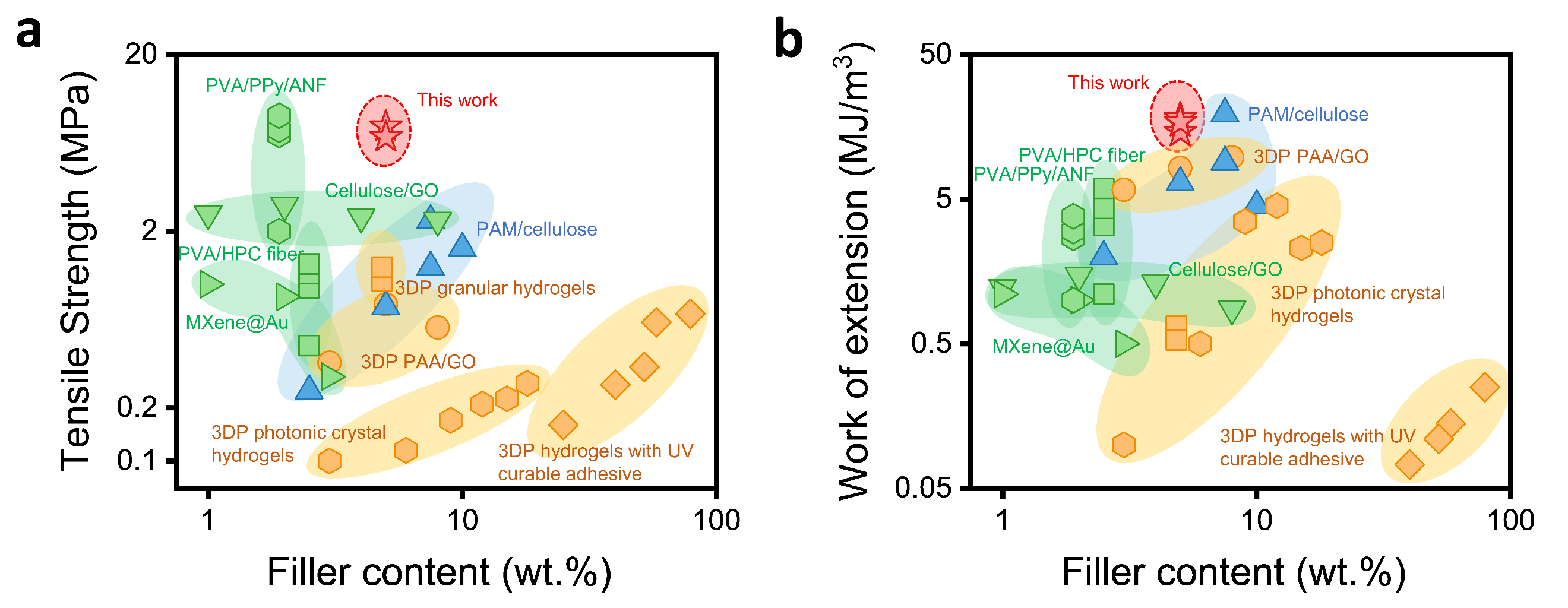
**

**Supplementary Fig. 8.** Comparison of the composite organo-hydrogels in this work with other composite hydrogels in the literature in terms of **a**) tensile strength and **b**) work of extension against filler content. See Supplementary Table 1 for a detailed list of data.


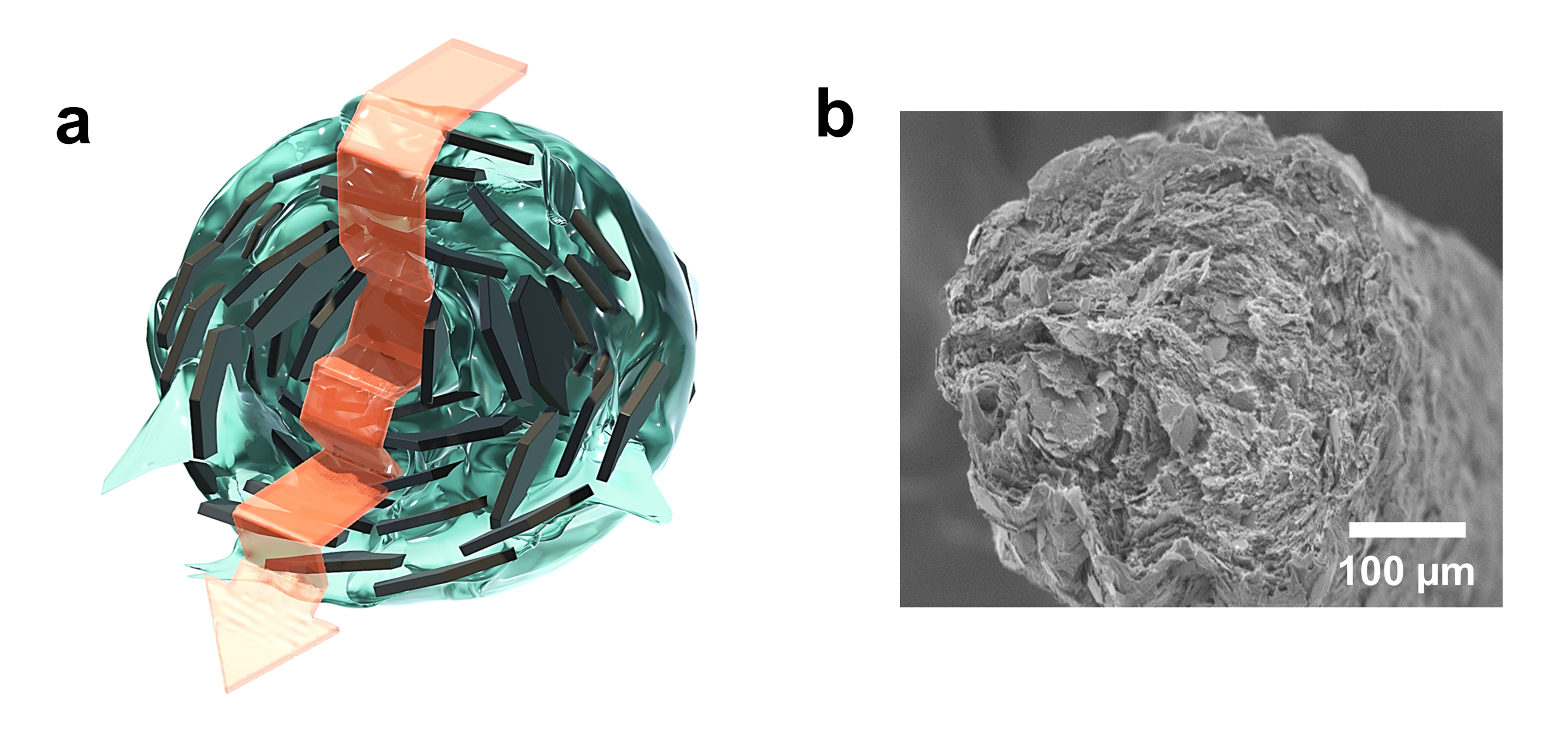
**Supplementary Fig. 9.** Illustration and SEM image of the fracture surface of a composite organo-hydrogel filament.

**
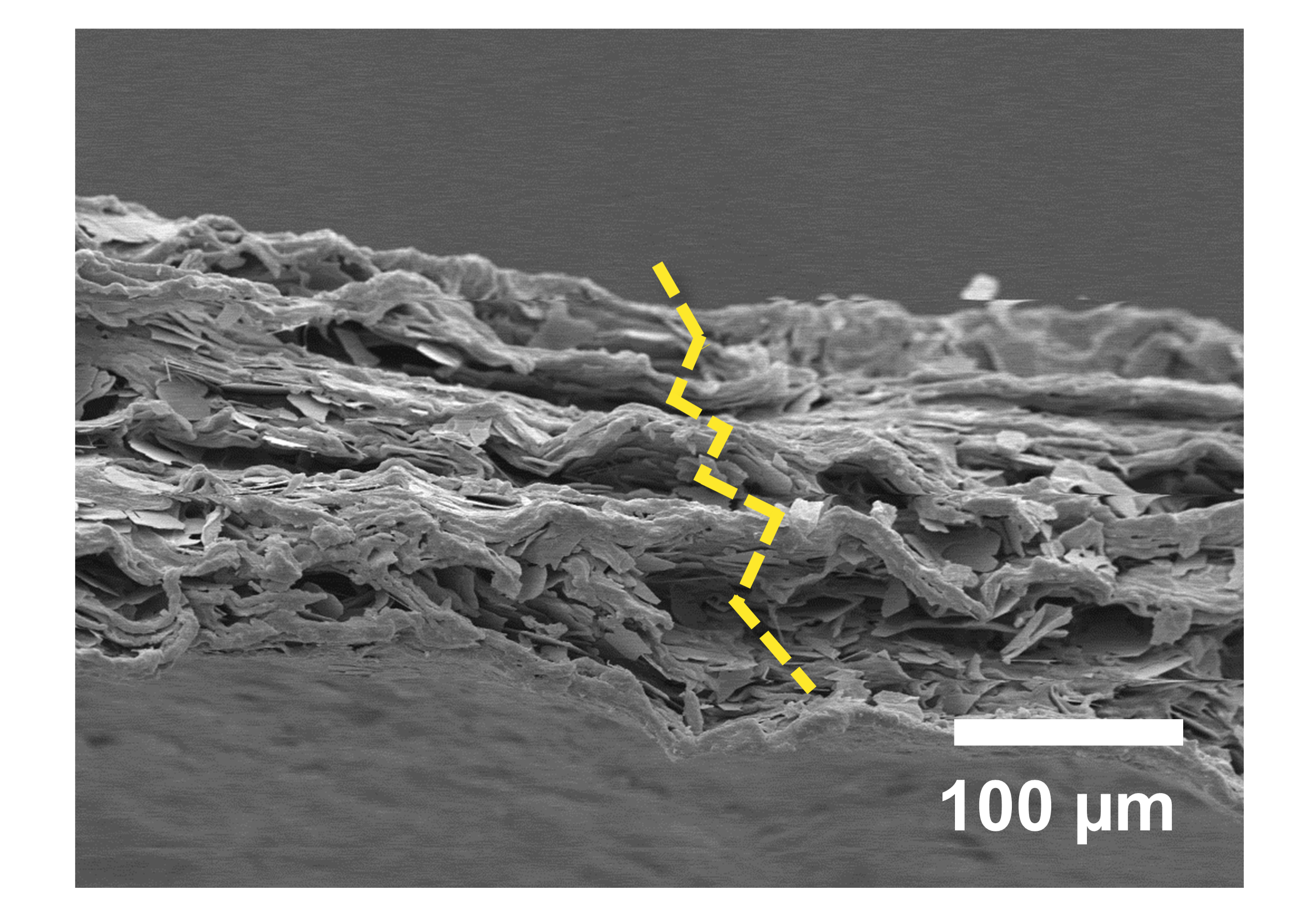
**

**Supplementary Fig. 10.** Fracture surface of composite organo-hydrogel sample with the Bouligand architecture.

**Supplementary Fig. 11.** Electrical conductivity of pure and composite organo-hydrogels with 2, 5 and 10 wt.% ceramic content. Data are presented as mean ± standard deviation from n=3 independent samples.

**Supplementary Fig. 12.** Thermal conductivity of pure and composite organo-hydrogels with 2, 5 and 10 wt.% ceramic content. Data are presented as mean ± standard deviation from n=3 independent samples.

**
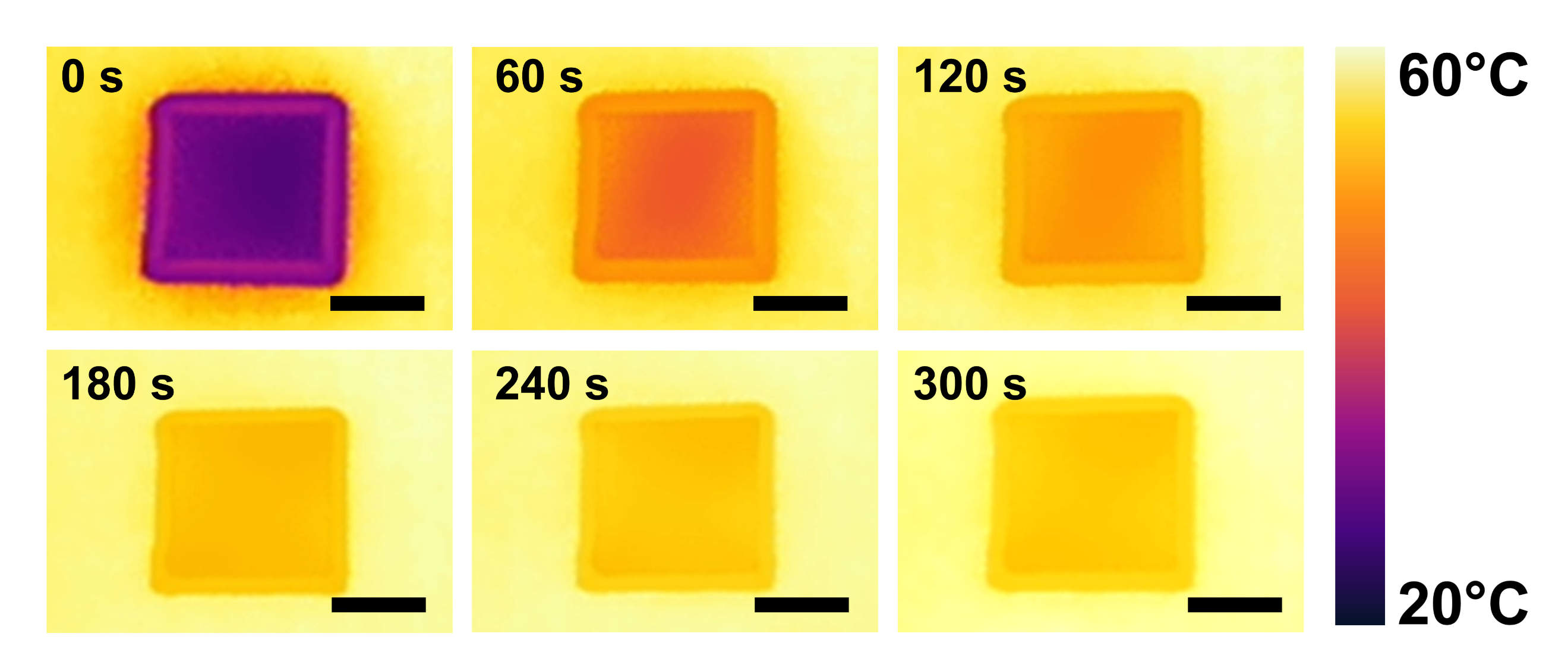
Supplementary Fig. 13.** Infrared images of a 5 wt.% composite organo-hydrogel on a heating plate at 60 °C. The scale bar is 1 mm.


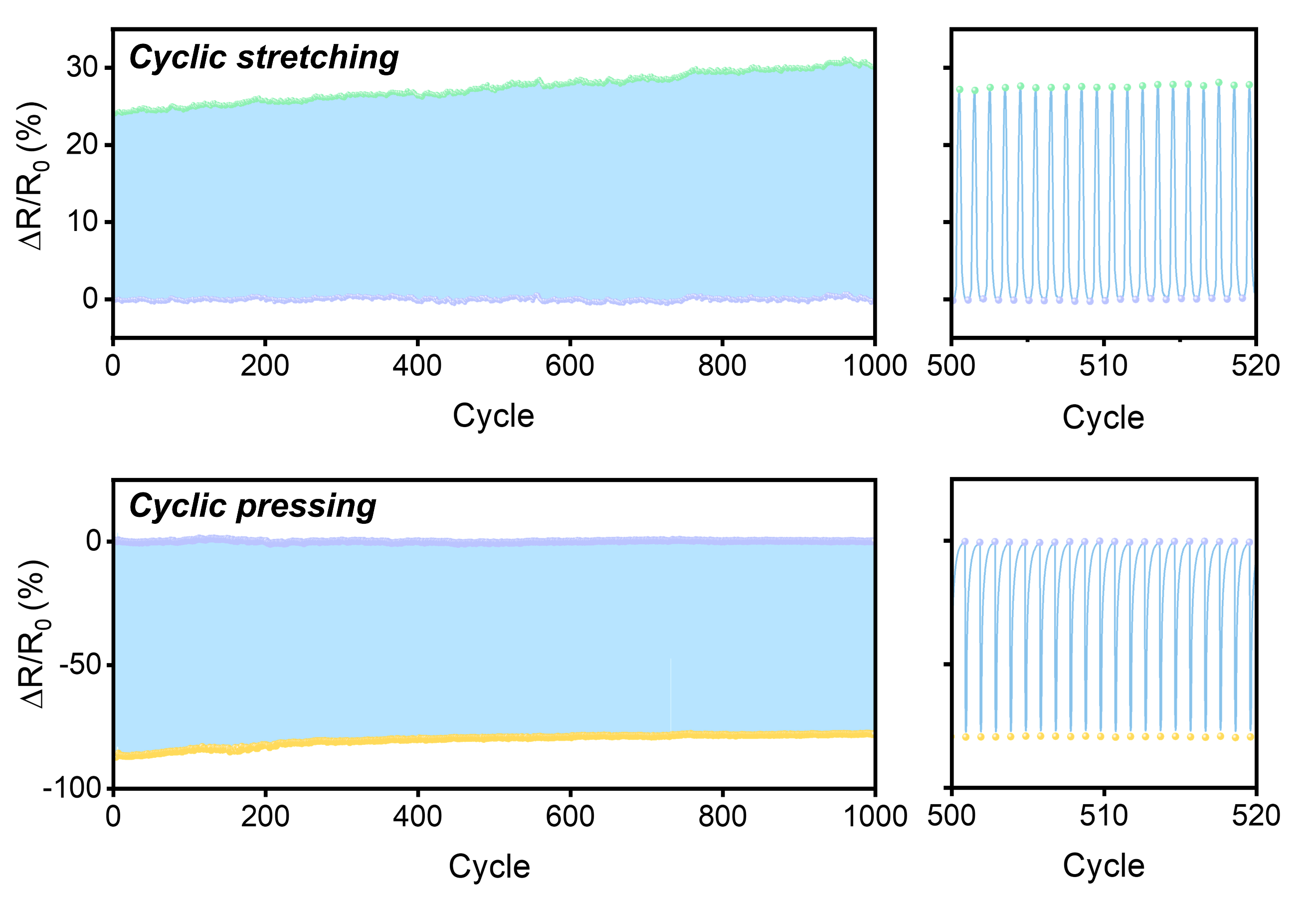


**Supplementary Fig. 14.** Resistance change signals of composite organo-hydrogels under cyclic stretching and pressing.

**Supplementary Table 1.** Mechanical properties of the composite organo-hydrogels in this work and (composite) hydrogels reported in the literature.

| Material | Filler content (wt.%) | Strain (%) | Young’s modulus (MPa) | Tensile strength (MPa) | Work of extension (MJ/m^3^) | Ref |
| --- | --- | --- | --- | --- | --- | --- |
| **This work** | **5** | **328 – 392** | **4.93 – 9.60** | **6.80 – 7.81** | **14.5 – 18.1** |  |
| Bacteria cellulose (BC) /MXene^1^ | 0.1 – 2 | 35 – 62 | 0.45 – 0.82 | – | – | 3. |
| MXene@Au hydrogels | 0.05 – 0.3 | – | – | 0.3 – 1.75 | 0.5 – 2.25 | 4. |
| Cellulose/graphene oxide (GO) | 1-8 | 17 – 107 | 0.09 – 2.2 | 0.02 – 2.8 | 0.01 – 1.48 | 6. |
| PVA/hydroxypropyl cellulose (HPC) fiber | 2.5 | 500 – 850 | 0.15 – 0.58 | 0.45 – 1.3 | 1.1 – 5.9 | 8. |
| Polyacrylamide (PAM)/cellulose | 2.5 – 10 | – | – | 0.25 – 2.3 | 2 – 19.5 | 9. |
| 3DP^2^ granular hydrogel | 4.83 | 120 – 130 | 0.48 – 0.57 | 1.05 – 1.25 | 0.53 – 0.66 | 44. |
| 3DP supramolecular hydrogel | 3 – 4 | 705 – 800 | 0.2 – 0.53 | – | – | 45. |
| 3DP hydrogels with UV curable adhesive | 25 – 81 | 38 – 75 | 0.35 – 2.9 | 0.09 – 1.1 | 0.019 – 0.66 | 46. |
| Mechanically trained PVA | – | 270 – 455 | 0.1 – 0.25 | – | – | 47. |
| Pre-stretched multilayer cellulose hydrogels | – | 25 – 150 | 4 – 110 | – | – | 48. |
| 3DP metal–organic framework (MOF) hydrogels | – | 290 – 453 | 0.135 – 0.15 | – | – | 49. |
| Polyampholyte hydrogels | – | – | – | 1.3 – 1.9 | 4.3 – 8.2 | 50. |
| 3DP photonic crystal hydrogels | 3 – 18 | – | – | 0.1 – 0.275 | 0.1 – 4.5 | 51. |
| Gelatin/sodium alginate (SA) double network hydrogels | – | – | – | 0.12 – 0.42 | 0.2 – 0.95 | 52. |
| 3DP polyacrylic acid (PAA)/GO | 3 – 8 | – | – | 0.36 – 0.77 | 5.8 – 9.7 | 53. |
| PVA/polypyrrole (PPy)/ aramid nanofiber (ANF) | 1.9 – 5 | 20 – 50 |  | 2 – 9 | 1 – 4 | 54. |

^1^Composite hydrogels are indicated as matrix phase/reinforcement phase.

^2^3DP: Abbreviation for 3D printed.
