# Supplementary material for: 3D printable strong and tough composite organo-hydrogels inspired by natural hierarchical composite design principles
Source: Nat Commun. 2024 Apr 15;15:3237. doi: 10.1038/s41467-024-47597-7 (PMC11018840; doi:10.1038/s41467-024-47597-7)
Supplement: Supplementary file 3 — Description of Additional Supplementary Files [file 41467_2024_47597_MOESM3_ESM.pdf]

Description of Additional Supplementary Files

File Name: Supplementary Movie 1
Description: Control of a robotic vehicle via a smart sensing glove.

File Name: Supplementary Movie 2
Description: Conductive fingertips for touch screen interaction.
